# Exposure to Radiofrequency Electromagnetic Fields From Wi-Fi in Australian Schools

**DOI:** 10.1093/rpd/ncw370

**Published:** 2017-01-10

**Authors:** Ken Karipidis, Stuart Henderson, Don Wijayasinghe, Lydiawati Tjong, Rick Tinker

**Affiliations:** 1 Australian Radiation Protection and Nuclear Safety Agency, 619 Lower Plenty Road, YallambieVIC 3085Australia

## Abstract

The increasing use of Wi-Fi in schools and other places has given rise to public concern that the radiofrequency (RF) electromagnetic fields from Wi-Fi have the potential to adversely affect children. The current study measured typical and peak RF levels from Wi-Fi and other sources in 23 schools in Australia. All of the RF measurements were much lower than the reference levels recommended by international guidelines for protection against established health effects. The typical and peak RF levels from Wi-Fi in locations occupied by children in the classroom were of the order of 10^−4^ and 10^−2^% of the exposure guidelines, respectively. Typical RF levels in the classroom were similar between Wi-Fi and radio but higher than other sources. In the schoolyard typical RF levels were higher for radio, TV and mobile phone base stations compared to Wi-Fi. The results of this study showed that the typical RF exposure of children from Wi-Fi at school is very low and comparable or lower to other sources in the environment.

## INTRODUCTION

The use of Wi-Fi technology has become increasingly common in many places throughout the community, including schools. Through the use of this technology, electronic devices are connected to a computer network wirelessly using radiofrequency (RF) electromagnetic fields, thereby eliminating or reducing the need for network cables in the classrooms and other places. A common example is a laptop or tablet connected to the internet via Wi-Fi access points installed around the school.

Wi-Fi is a type of wireless local area network which operates in unlicensed regions of the RF spectrum in the 2.45 and 5 GHz bands. The technology is designed to be used up to a few tens of metres between a device and an access point. Over these short distances Wi-Fi devices only use low output power, typically limited to 2 W or less. Children in a Wi-Fi enabled school are exposed to low level RF fields intermittently when using devices on the network and also from the access points and some portion of the transmitted RF energy is absorbed within their bodies^(1)^.

The increasing popularity of Wi-Fi technology^(2)^ has given rise to public concern about the RF exposure from Wi-Fi equipment, particularly in schools. Some individuals and groups including parents have publicly expressed concern that RF exposure from the technology has the potential to adversely affect children as well as the general population. Moreover, there are groups of concerned citizens actively campaigning against the installation and use of wireless technologies in schools and other public places^(3)^.

International exposure guidelines for RF fields have been developed on the basis of current scientific knowledge to ensure that RF exposure is not harmful to human health^(4, 5)^. The guidelines developed by the International Commission on Non-Ionizing Radiation Protection (ICNIRP) in particular form the basis for regulations within most parts of the European Union and many other countries including Australia^(6)^. The exposure limits in the ICNIRP guidelines, which include basic restrictions and indicative reference levels for measurement, are intended to protect people of all ages and health status against all established adverse health effects that result from excessive RF exposure.

A limited number of previous measurement surveys have shown that exposure to RF fields from Wi-Fi in public places is expected to be much lower than the reference levels for public exposure specified in the ICNIRP guidelines^(7–9)^. Although Wi-Fi clearly operates at low power, little data is currently available on typical RF exposures from wireless networks in schools^(1, 10)^. It is therefore important to measure the RF exposure of children from Wi-Fi in schools and compare it with the ICNIRP exposure guidelines, but also with exposures from other common sources of RF in the environment.

In the present study the Australian Radiation Protection and Nuclear Safety Agency (ARPANSA) conducted measurements of RF electromagnetic fields from Wi-Fi and other sources in 23 schools located in two states in Australia. The main aims of the study were to measure the typical and peak RF exposure from Wi-Fi in the classroom and schoolyard and compare these against the public exposure reference levels of the ICNIRP guidelines. In order to better understand the RF exposure environment in these schools, the Wi-Fi measurement results were also compared to RF exposure from other sources in the everyday environment, such as mobile phone base stations, radio and TV towers and other sources.

## METHODS

### Selection and recruitment of schools

Schools were selected from the two most populated states in Australia, New South Wales (NSW) and Victoria. ARPANSA initially engaged with the education departments in Victoria and NSW in order to seek permission to conduct the study and acquire a list of possible schools that can be invited to participate in each state. The Victorian Department of Education and Training provided a list of 220 schools in Victoria with multiple Wi-Fi access points. The list of Victorian schools was classified into 10 groups consisting of a mixture of metro and rural schools, secondary and primary schools, smaller and larger schools (according to student numbers), and schools with a small and large number of access points. From each of the 10 groups one school was randomly selected to be invited to participate. The NSW Department of Education provided a list of 17 schools that self-nominated based on a bulletin about the study that was circulated to all NSW schools by the Department.

The 10 Victorian and 17 NSW schools were invited to participate in the study during June 2016. The letter of invitation included an information pack explaining the reasons for the study and a summary of the measurement protocol. Initially three Victorian and 10 NSW schools agreed to participate in the study. A further 53 Victorian schools were selected and invited to participate during June-August 2016; of these, nine agreed to participate. The NSW Department of Education in July 2016 requested that another school which had experienced parental concerns regarding Wi-Fi at the school be included in the study. In total 23 schools, 12 in Victoria and 11 in NSW participated in the study. Different characteristics of the schools are shown in Table 1.

**Table 1.** Different characteristics of the 23 schools that participated in the study.

	Number of schools
Type of school	
Primary	7
Secondary	16
Location	
Metro	18
Rural	5
Number of students	
<600	6
600–1000	9
>1000	8
Number of access points^a^	
<40	6
40-–70	11
>70	6

^a^Information on the number of access points was not provided by one school and was estimated using linear regression between number of students and number of access points.

### Measurements

The participating schools were visited for measurements during June to September 2016. All measurements were performed via appointment mainly during school hours between 8.30 am and 3.30 pm; one school was measured during school holidays and another during the school's sports day where all the students were off campus. All the measurements were performed by technically trained ARPANSA staff members.

RF fields were measured using a calibrated Narda SRM–3006 Selective Radiation Meter (Narda Safety Test Solutions, NY, USA; traceable in accordance with ISO/IEC 17025) and three separate tri-axial probes (one magnetic and two electric field probes) covering different frequency ranges from 9 kHz to 6 GHz. The meter was set to record the power flux density of the RF field (in units of W/m^2^).

At each school, the measurements were conducted in one classroom equipped with a Wi-Fi access point and one outdoor location in the schoolyard which were selected in consultation with the school principal/representative; in two schools the classroom measurement was performed in the library which was often used as a classroom. For the majority of schools (20) the measurements were conducted in an empty classroom to avoid lesson disruption; in two schools the classroom was measured with students present at the request of the principal and in another the classroom was measured with a group of teachers present that were preparing a lesson plan.

The measurements included recording the average and maximum RF fields due to Wi-Fi whilst moving throughout the classroom; detailed measurements of the specific frequency bands used by Wi-Fi technologies at several stationary positions within the classroom; and recording all detectable RF signals up to 6 GHz, representing different RF sources, in the classroom and in the schoolyard.

#### Classroom walk-through Wi-Fi measurements

Spatial measurements of RF fields from Wi-Fi were recorded whilst walking slowly throughout the classroom and sweeping the probe slowly up and down (up to head height) over a period of 10 min (idle mode). All readily accessible locations within the classroom were visited at least once, paying particular attention to student desks and locations close to the nearest Wi-Fi access point. This procedure was repeated whilst downloading (or uploading) large files, browsing the internet or otherwise interacting with the Wi-Fi using one or more laptops in the classroom (active mode). During both the idle and active mode walk-throughs the average and maximum RF fields over the ten-minute period were recorded representing the typical and peak exposure in locations usually occupied by students in the classroom.

#### Classroom stationary Wi-Fi measurements

Measurements of RF fields from Wi-Fi were conducted at 1.5 m above the ground (representing the head/torso of a child), with the probes mounted on a tripod, at stationary locations in the classroom for one minute whilst the Wi-Fi was active. The stationary locations included:

- Nominal centre of the classroom.
- Nearest student desk to access point.
- Furthest student desk to access point.
- At the access point, either directly underneath a ceiling mounted access point or 0.5 m from the wall of a wall mounted access point.

The average and maximum RF fields from Wi-Fi over the 1-min period for each location were recorded.

#### Measurements of all RF sources

At the nominal centre of the classroom, RF fields were measured at 1.5 m above the ground for 1 min in various frequency bands across the spectrum representing different RF sources including (AM and FM) radio, TV, mobile telephone base stations (downlink only), Wi-Fi and other sources; these are listed in Table 2. The average and maximum RF fields over the 1-min period for each frequency band were recorded. These measurements were repeated at the chosen location in the schoolyard of each school. The schoolyard measurements were conducted in open areas which were at least 5 m clear of buildings, playground equipment, trees or bushes or other physical obstructions.

**Table 2.** The frequency ranges for the different RF sources that were measured in the study.

RF source	Type	Frequency range
Radio	AM	526.5 kHz–1.6065 MHz
	FM	87.5–108 MHz
	DAB	202–209 MHz
TV	VHF TV	174–202 MHz
	VHF TV	209–230 MHz
	UHF TV	526–820 MHz
Mobile		758–788 MHz
		870–890 MHz
		935–960 MHz
		1.805–1.88 GHz
		1.9–1.92 GHz
		2.11–2.17 GHz
		2.302–2.4 GHz
		2.57–2.62 GHz
		2.62–2.69 GHz
		3.425–3.575 GHz
Wi-Fi		2.4–2.5 GHz
		5.15–5.85 GHz
Other	VHF Paging	148–174 MHz
	UHF Paging	403–420 MHz
	UHF Paging	450–520 MHz
	ISM	915–928 MHz
	DECT	1880–1900 MHz

### Statistical analysis

The RF levels that were measured are presented both as power flux density values and as percentages of the power flux density reference levels for the general public recommended in the ICNIRP Guidelines. Depending on the frequency of the RF source, the ICNIRP reference levels vary from 2 to 10 W/m^2^; for Wi-Fi the ICNIRP reference level is 10 W/m^2^^(4)^.

Descriptive statistics were calculated for all the measurements. Measurement distributions were tested for normality using the Shapiro–Wilk test. Comparisons between idle and active Wi-Fi measurements were tested for statistical significance using the Wilcoxon signed-rank test. Comparisons between measurements of Wi-Fi at different locations in the classroom and Wi-Fi compared to other RF sources in the classroom and schoolyard were tested for statistical significance using the Mann–Whitney test.

The effect of different school characteristics (type of school, metro or rural location, number of students, number of access points) on RF levels from Wi-Fi were investigated using multivariate linear regression. All the analyses were performed with the SPSS software (SPSS Inc., Chicago, IL, USA; version 23.0) and the level of significance was set at an *α*-level of 0.05.

## RESULTS

### Measurements

All the RF levels measured in the 23 schools were much lower than the exposure reference levels of the ICNIRP Guidelines. The measurements showed a lognormal distribution (*p* < 0.01 for all) so they are better described by nonparametric statistics.

#### Classroom walkthrough Wi-Fi measurements

**Figure 1.**
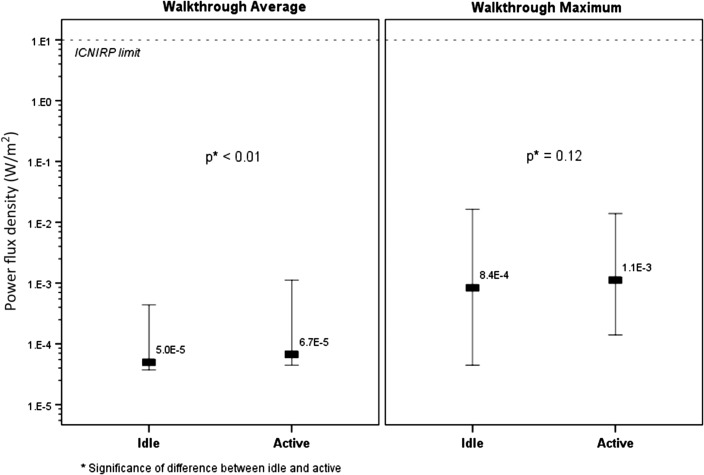
RF levels (indicating minimum, maximum and median) for the walkthrough Wi-Fi measurements taken in the classroom of the 23 schools under the idle and active conditions.

The RF levels for the walkthrough Wi-Fi measurements taken in the classrooms of the 23 schools under the idle and active conditions are shown in Figure 1. The median of the walkthrough average in all the schools (typical exposure) was only slightly higher when the Wi-Fi in the classroom was active (7 × 10^−4^% ICNIRP reference level) compared to when it was idle (5 × 10^−4^%); significance of difference between the idle and active walkthrough averages was *p* < 0.01. There was no statistically significant difference between the walkthrough maximum in all the schools (peak exposure) when the Wi-Fi in the classroom was active compared to idle (*p* = 0.12).

#### Classroom stationary Wi-Fi measurements

**Figure 2.**
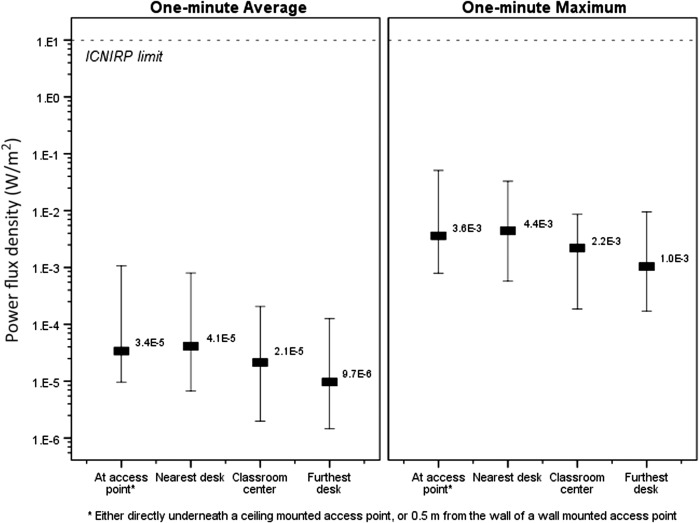
RF levels (indicating minimum, maximum and median) for the stationary Wi-Fi measurements taken at different locations in the classroom of the 23 schools.

The RF levels for the stationary Wi-Fi measurements taken at different locations in the classroom are shown in Figure 2. The RF levels at the furthest desk to the access point (median distance 6.8 m) were slightly lower than the nearest desk to the access point (1.9 m); the medians for the typical exposure (1-min measurement average) and peak exposure (1-min measurement maximum) were 10^−4^ and 10^−2^% of the ICNIRP reference level, respectively at the furthest desk compared to 4 × 10^−4^ and 4 × 10^−2^% at the closest desk (*p* < 0.01 for both the typical and peak exposure comparisons). The RF levels were similar between measurements conducted next to/under the access point (1.3 m) and the nearest desk to the access point (*p* > 0.5 for both the typical and peak exposure comparisons).

#### Measurements of all RF sources

**Figure 3.**
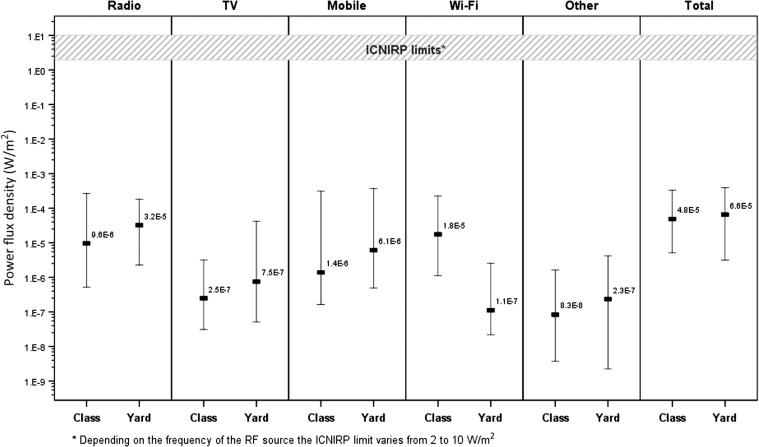
Typical (1-min average) RF levels for the measurements of all RF sources taken in the centre of the classroom and in the schoolyard.

**Figure 4.**
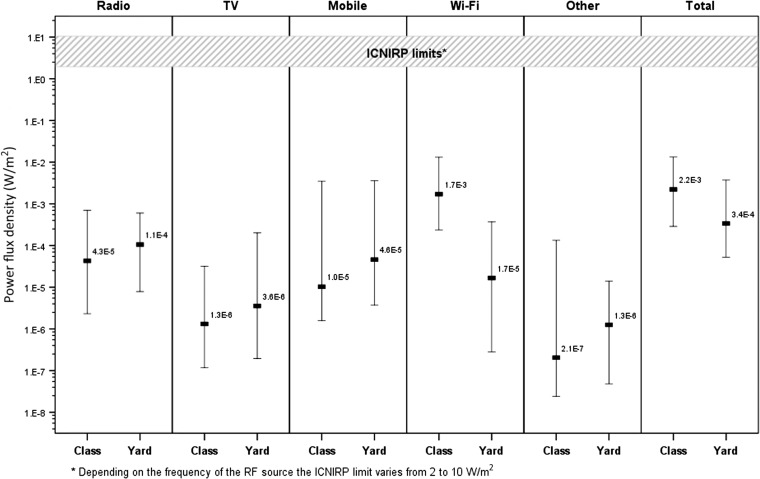
Peak (1-min maximum) RF levels for the measurements of all RF sources taken in the centre of the classroom and in the schoolyard.

The RF levels for the measurements of all RF sources taken in the centre of the classroom and in the schoolyard are shown in Figures 3 and 4 for typical (1-min measurement average) and peak (1-min measurement maximum) exposure, respectively. In the classroom the typical RF levels were similar between Wi-Fi and radio (*p* = 0.46) but higher compared to other sources (*p* < 0.01 for all comparisons). The peak RF levels in the classroom were higher for Wi-Fi compared to all other sources, including radio (*p* < 0.01 for all) and also higher than all other sources combined (*p* < 0.01).

In the schoolyard the typical RF levels due to radio, TV and mobile phone base stations were higher compared to Wi-Fi (*p* < 0.01 for all). Similarly the peak RF levels in the schoolyard were higher due to radio and mobile phone base stations compared to Wi-Fi (*p* < 0.02 for both) but Wi-Fi was higher than TV (*p* < 0.01).

For the total RF levels from all sources the typical exposure was similar between the classroom and schoolyard (*p* = 0.98) but the peak exposure was higher in the classroom compared to the schoolyard (*p* < 0.01), mainly due to the maximum Wi-Fi signal.

### Effect of schools characteristics

The multivariate linear regression showed that none of the school characteristics had an effect on the measured RF levels from Wi-Fi (*p* > 0.05 for all).

## DISCUSSION

The current study was one of the most comprehensive surveys ever conducted measuring RF electromagnetic fields from Wi-Fi and other sources in schools. It employed extensive measurements including a walkthrough survey (spatial measuremnts) in a classroom; stationary measurements of Wi-Fi at different locations in the classroom and measurements of all RF sources in the classroom and the schoolyard.

All the RF levels measured in this study were much lower than the exposure reference levels for the general public recommended by the ICNIRP guidelines^(4)^. The average (typical) and maximum (peak) RF levels from Wi-Fi in locations occupied by students in the classroom were of the order of 10^−4^ and 10^−2^% of the ICNIRP reference level, respectively. This was expected given the low output power of Wi-Fi equipment and measurement data from previous studies. Typical exposure to Wi-Fi in public places was also found to be well within the ICNIRP guidelines by Schmid *et al*.^(7)^, Foster^(8)^ and more recently by Industry Canada^(9)^. Specifically investigating schools, Peyman and colleagues^(1, 10)^ found maximum RF levels due to Wi-Fi in the order of 10^−2^% of the ICNIRP reference levels at distances of 1–2 m from the access point. More recently, Gledhill^(11)^ measured RF levels in different classroom locations of two schools and found average and maximum levels of less than 10^−2^% and 3 × 10^−2^% of the ICNIRP reference levels, respectively.

In the current study the 10-min walkthrough survey in the classroom showed that the typical RF levels were only slightly higher when the Wi-Fi in the classroom using one or more laptops was active compared to when it was idle. However there was no difference in the peak RF levels when the Wi-Fi in the classroom was active compared to idle. The access point regularly transmits short duration beacon signals to enable client devices (e.g. laptops) to identify and synchronise with the network. These beacon signals are transmitted at full power even when no device is connected (idle mode). Additional bursts are transmitted from the access point when communicating with a connected device (active mode). In the active mode the access point is sending signals more often but not at any higher power than in idle mode. Hence, there is no difference in the maximum detected signal between active and idle mode, but the average measured signal is higher in the active mode compared to the idle mode.

For the stationary Wi-Fi measurements conducted in the classroom the current study showed that the RF levels were slightly higher in the nearest desk to the access point compared to the furthest desk. This was expected since RF decreases with the inverse-square of the distance and assuming that there is no other access points present in close distance. A similar pattern of decreasing RF levels with increasing distance from the access point was shown by Peyman *et al*.^(1)^ and Gledhill^(11)^. Interestingly, in our study there was no significant difference in the RF levels measured between next to/under the access point and the nearest desk to the access point. Although the positioning of the access point in the classroom of the 23 schools varied quite substantially it was often quite close to the nearest desk.

Comparing Wi-Fi to other RF sources, the current study showed that the typical RF levels in the classroom were similar between Wi-Fi and radio (in the order of 10^−4^% ICNIRP reference level). The peak exposure was higher for Wi-Fi compared to other sources and this was due to the beacon signal from the Wi-Fi transmitting at full power as explained earlier. The measurements conducted in the schoolyard showed that the typical RF levels from other sources such as radio, TV and mobile phone base stations were higher compared to Wi-Fi. Access points in schools are mainly installed for indoor coverage. A previous survey conducted by ARPANSA measured RF levels from different sources at 41 outdoor locations across Melbourne, Australia^(12)^. This previous study also showed that the RF levels from broadcast antennas and mobile phone base stations were higher compared to Wi-Fi. Similarly Joseph *et al*.^(13, 14)^ showed that average RF levels measured in five European countries from various sources in different urban settings (outdoor, offices, public transport and homes) were generally higher from broadcast and mobile telephony transmissions compared to Wi-Fi.

The current study showed that none of the school characteristics had an effect on the measured RF levels from Wi-Fi. It was expected that the type (primary/secondary) and location (metro/rural) of the school would not have an influence on the results. There was a linear correlation between number of students and number of access points which was expected given that a larger school would require more access points to service its campus. This study showed that having more students and more access points does not have a major influence on the personal exposure of each student to Wi-Fi which will be largely dominated by the closest access point or client device rather than the total number of access points around the school.

Wi-Fi transmissions consist of sequences of RF burst signals or pulses ranging in duration depending on the amount of data being carried by a pulse^(15)^. The proportion of time that Wi-Fi transmits RF signals is called the duty cycle. Joseph *et al*.^(14)^ in measuring Wi-Fi in 176 different urban locations (outdoors, homes, offices) found a median duty cycle of 1.4% over all the measurements. Particularly in schools, Khalid *et al*.^(10)^ in measuring Wi-Fi in six schools found a mean duty cycle from the access points of 4.8%. In our study duty cycle was measured separately for the 2.45 and 5 GHz transmissions when performing the stationary Wi-Fi measurements in the centre of the classroom. The median duty cycle for 23 schools that were measured in the current study was 6.3 and 2.4% for 2.45 and 5 GHz transmissions, respectively.

Members of the public often ask about the cumulative exposure that a child receives when using a Wi-Fi device in a classroom in which a number of children are simultaneously using Wi-Fi. When downloading files, most of the transmissions will be from the access point, not the students’ device. When downloading and uploading only a portion of the maximum capacity of a network would be used even in a classroom filled with children using Wi-Fi. The Wi-Fi network divides RF transmissions among the access points and client devices therefore the individual RF exposure to a child in a classroom that is using a device consists of sequential exposures from all active devices, the majority of which are located at some distance away^(15)^. For the majority of schools (20) the measurements in the current study were conducted in an empty classroom (to avoid lesson disruption) with an access point and one laptop. In three schools, measurements were conducted with students or teachers present and using Wi-Fi devices. A comparison between measurements conducted in empty classrooms and classrooms with multiple students/teachers using Wi-Fi showed no significant difference in the RF levels (*p* > 0.1 for all); although this may have been due to low numbers (only three schools measured with multiple users in the classroom).

The results of this study showed that children's exposure to RF fields from Wi-Fi in schools is several orders of magnitude below exposure reference levels recommended by international guidelines for protection against established health effects. Further, the exposure from Wi-Fi is typically comparable or lower to other common sources in the environment.
